# ERBB2/HER2 mutations are transforming and therapeutically targetable in leukemia

**DOI:** 10.1038/s41375-020-0844-7

**Published:** 2020-05-04

**Authors:** Sunil K. Joshi, Jamie M. Keck, Christopher A. Eide, Daniel Bottomly, Elie Traer, Jeffrey W. Tyner, Shannon K. McWeeney, Cristina E. Tognon, Brian J. Druker

**Affiliations:** 1 Knight Cancer Institute, Oregon Health & Science University, Portland, OR USA; 2 Department of Physiology & Pharmacology, School of Medicine, Oregon Health & Science University, Portland, OR USA; 3 Division of Hematology & Medical Oncology, Department of Medicine, Oregon Health & Science University, Portland, OR USA; 4 Center for Spatial Systems Biomedicine, Oregon Health & Science University, Portland, OR USA; 5 Department of Cell, Development, & Cancer Biology, Oregon Health & Science University, Portland, OR USA; 6 Division of Bioinformatics and Computational Biology, Department of Medical Informatics and Clinical Epidemiology, Oregon Health & Science University, Portland, OR USA

**Keywords:** Acute lymphocytic leukaemia, Acute myeloid leukaemia

## To the Editor:

The ErbB/HER family of receptor tyrosine kinases consists of four cell surface glycoproteins: epidermal growth factor 1 (EGFR or ErbB1), ErbB2 (c-Neu or human EGF receptor 2 [HER2]), ErbB3, and ErbB4 [1, 2]. Binding of the soluble ligand to its cognate ErbB receptor induces formation of homo- or heterodimer complexes, which activates receptor tyrosine kinase activity, leading to increased downstream RAS/MAPK, PI3K/AKT, and JAK/STAT signaling [1].

These receptors play a fundamental role in the development, proliferation, and differentiation of epithelial, mesenchymal, and neuronal tissues; however overexpression, amplification, and activating point mutations of these receptors promote oncogenesis [1]. ErbB2 amplification or overexpression is observed in ~25–30% of breast cancers [1] and is associated with an aggressive clinical phenotype. In vitro studies have shown that ErbB2/ErbB3 heterodimers act as an oncogenic unit to induce breast cancer cell proliferation via PI3K/AKT signaling [3]. Moreover, activating point mutations such as ERBB2^L755P^ and ERBB2^L869R^ enable ErbB2/ErbB3 dimerization, oncogenic signaling, and cell growth while the gatekeeper mutation ERBB2^T798I^ confers resistance [4, 5]. Activating EGFR kinase domain mutations have also been reported in non-small cell lung cancer (NSCLC) [6]. These mutants also dimerize with ErbB3, culminating in upregulation of PI3K/AKT signaling and aberrant cell proliferation.

Despite the well-studied role of ErbB receptors in solid tumors and paralleled advances in their therapeutic targeting, the potential of deregulated ErbB signaling to contribute to leukemia is largely unknown. We recently performed deep sequencing on primary samples from patients with a range of hematologic malignancies and discovered point mutations in the ErbB2 receptor in a small subset of patients. Here, we show that these mutations are oncogenic and cells transformed by these mutations are sensitive to several irreversible ErbB inhibitors and trastuzumab.

Point mutations in the ERBB2 gene were uncovered and characterized in three of 185 patients with hematologic malignancies following deep sequencing using a custom capture library consisting of 1862 kinase and kinase-associated genes. The cohort included patients with acute myeloid leukemia (AML; *n* = 96), acute lymphoid leukemia (ALL; *n* = 51), and myeloproliferative neoplasms (MPN; *n* = 38). The three ERBB2 mutations were prioritized by the HitWalker algorithm [7], which uses ex vivo functional drug screening data collected in parallel to rank mutations that may be associated with critical survival pathways in a given patient sample (Supplementary Fig. 1).

**Fig. 1:**
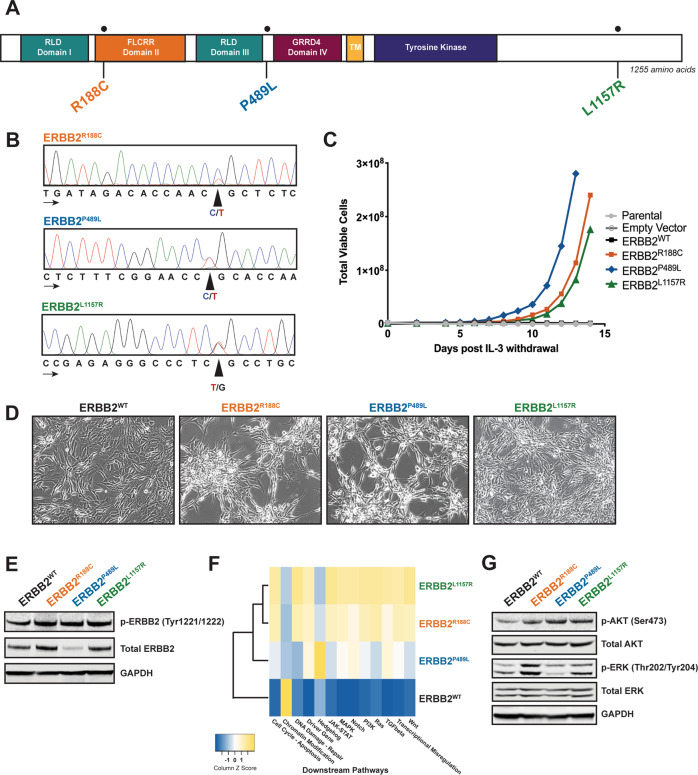
ERBB2 point mutations found in leukemia samples are oncogenic, enable cytokine-independent proliferation, and downstream activation. **a** ERBB2 gene schematic with location of point mutations depicted. The location of the following domains is included: receptor L domain (RLD, domains I and III), furin-like cysteine rich region (FLCRR, domain II), growth factor receptor domain (GRRD4, domain IV), transmembrane domain (TM), and tyrosine kinase domain. **b** Sanger sequencing chromatograms of patient genomic DNA confirm the presence of ERBB2^R188C^, ERBB2^P489L^, and ERBB2^L1157R^ mutations. Peaks correspond to the following nucleotides: A (green), T (red), C (blue), and G (black). Arrows indicate direction of sequencing. **c** ERBB2^R188C^, ERBB2^P489L^, and ERBB2^L1157R^ mutations transform the murine Ba/F3 pro-B cell line to IL-3-independent growth. No growth was observed in parental Ba/F3 cells or cells harboring an empty vector or wild-type (WT) ERBB2. All engineered Ba/F3 cell lines were flow sorted for low, equivalent GFP expression prior to the IL-3 withdrawal assay. Total viable cells are plotted over time after the withdrawal of IL-3. This experiment was repeated at least twice with consistent results. **d** Stable expression of ERBB2 mutants in NIH 3T3 fibroblasts exhibited spontaneous foci formation in monolayers independent of cell plating density. 3T3 cells transduced with ERBB2^WT^ lack this phenotype but exhibit an increased rate of cell growth. This experiment was repeated at least twice with consistent results. **e** Immunoblot analysis of ERBB2-transformed Ba/F3 cells shows increased phosphorylation of ErbB2 with all three mutants compared with ERBB2^WT^. GAPDH served as a loading control. Prior to lysis, WT cells were grown in IL-3 supplemented media and all lines were starved overnight in 0.1% BSA RPMI. **f** In comparison with Ba/F3 cells expressing ERBB2^WT^, changes in gene expression were observed in mutant-transformed Ba/F3 cells. Most notably, all mutants resulted in an increase in expression of MAPK signaling. Prior to RNA isolation, WT cells were grown in IL-3 supplemented media and all lines were starved overnight in 0.1% BSA RPMI. Expression analysis was performed in triplicate. **g** Expression of phosphorylated AKT is increased in mutant-transformed Ba/F3 cells relative to WT cells. ERK phosphorylation was evident only in Ba/F3 cells expressing ERBB2^R188C^ and ERBB2^L1157R^. GAPDH served as a loading control. As noted above, all cell lines were starved overnight in 0.1% BSA RPMI.

Two mutations, ERBB2^R188C^ and ERBB2^P489L^, were found in pediatric patients with AML and ALL, respectively. Both mutations lie within the extracellular domain of ErbB2. Specifically, R188C resides within the furin-like cysteine rich region (FLCRR, domain II) and P489L is located in between the receptor L domain (RLD, domain III) and the growth factor receptor domain (GRRD4, domain IV). Another mutation discovered in an adult patient with AML, ERBB2^L1157R^, is located near the C-terminus (Fig. 1a, Supplementary Fig. 2). All three mutations were confirmed via Sanger sequencing (Fig. 1b). While P489L has been previously reported in patients with breast [8] and lung squamous cell carcinoma [9], to our knowledge, R188C and L1157R have not yet been reported. Due to the unavailability of a matched skin biopsy, we were unable to confirm if these mutations are somatic or germline. Information about patient variant allele frequency (VAF) and HitWalker ranking [7] is provided in Supplementary Table 1.

Given that very little is known about ERBB2 in hematopoiesis and leukemia, we sought to measure the expression of ERBB2 in available patient samples harboring the specific mutations discussed above. While no clinical material was available for the patient with ERBB2^L1157R^, we found that ERBB2 is expressed at the RNA level in both pediatric patients with ERBB2^R188C^ and ERBB2^P489L^ mutations via Affymetrix exon microarray analysis (Supplementary Fig. 3A, B).

To determine if the identified ERBB2 mutations induce oncogenic transformation, we stably expressed each of them in Ba/F3 cells, a murine IL-3-dependent pro-B cell line that provides a well-established model to PMID: 32315394. Oncogenes expressed in Ba/F3 cells enable IL-3 independent growth. Since it is known that overexpression of wild-type (WT) ERBB2 itself confers some transforming activity [4], we sorted all ERBB2 Ba/F3 cell lines for equivalent, low GFP-positive expression prior to performing the IL-3 withdrawal assay. At low expression, all three ERBB2 mutations enabled IL-3-independent growth and proliferation, whereas parental Ba/F3 cells or cells expressing empty vector or WT ERBB2 were unable to grow without IL-3 (Fig. 1c). Retroviral transduction of ERBB2 constructs into murine NIH 3T3 fibroblast cells further showed a transformed morphological phenotype and increased ability to spontaneously form foci when grown to confluence for ERBB2-mutant-expressing NIH 3T3 cells compared with those expressing ERBB2 WT or empty vector controls (Fig. 1d).

Using western blotting, we validated the expression of total and phosphorylated ErbB2 in our ERBB2-mutant-transformed Ba/F3 cell lines and found that all three mutants resulted in increased ErbB2 receptor phosphorylation at Tyr1221/1222 compared with WT (Fig. 1e). We also used these stable Ba/F3 cell lines to assess downstream signal transduction pathways that could contribute to the tumorigenic phenotype. With NanoString’s nCounter technology, we saw that ERBB2^R188C^ and ERBB2^L1157R^ resulted in upregulated gene expression of several pathways relative to ERBB2^WT^ cells, including JAK/STAT, PI3K/AKT, RAS/MAPK, and WNT signaling. Notably, with the ERBB2^P489L^ mutant, we saw an increase in hedgehog signaling (Fig. 1f). Similar to our NanoString data, we saw variability in downstream signaling among the mutants via western blotting (Fig. 1g). Together, these data indicate that these mutations in ERBB2 result in hyperactivation of the receptor with pursuant cellular transformation.

**Fig. 2:**
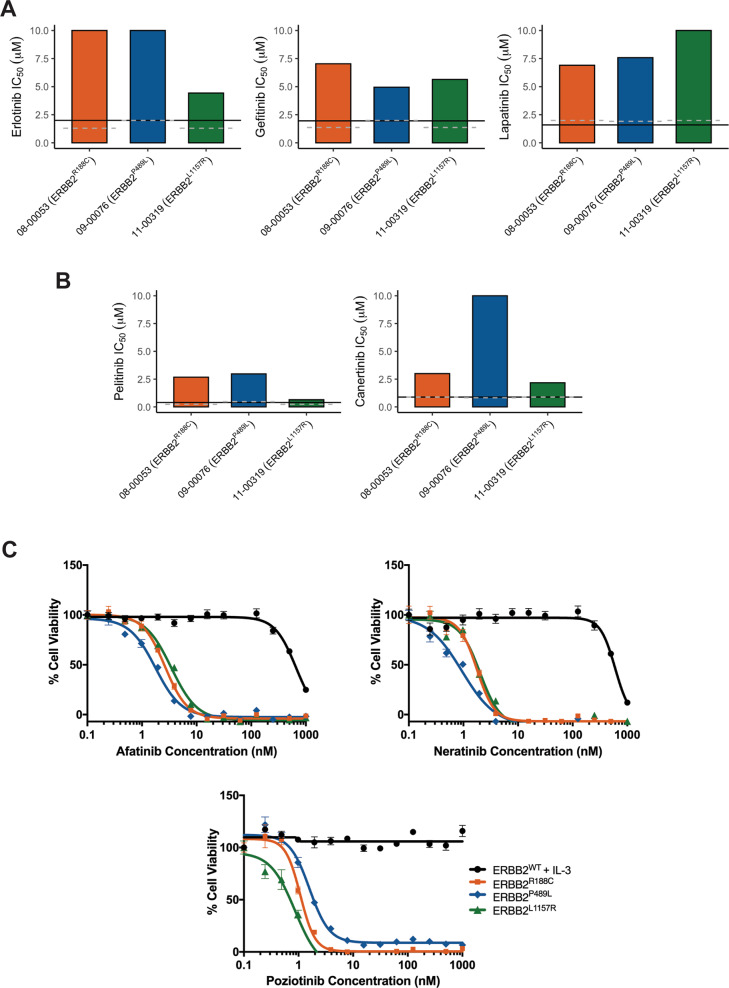
ERBB2 point mutations confer sensitivity to irreversible ErbB inhibitors. Ex vivo inhibitor profiles for patients 08-00053 (ERBB2^R188C^), 09-00076 (ERBB2^P489L^), and 13-00319 (ERBB2^L1157R^) with the IC_50_ measure of response to each reversible (**a**; erlotinib, gefitinib, and lapatinib) and irreversible (**b**; pelitinib and canertinib) inhibitor shown on the *Y* axis. Sensitivity is determined by % median IC_50_, which has historically been a marker for patient samples remarkably sensitive to a screened inhibitor [10]. Solid black lines indicates 20% of median IC_50_ for the overall cohort while the gray dotted line indicates 20% of the median IC_50_ for samples with similar diagnoses (i.e., acute myeloid leukemia or acute lymphoid leukemia). **c** ERBB2-mutant-transformed Ba/F3 cells are sensitive to irreversible inhibitors. Five replicates of WT and mutant ERBB2 Ba/F3 cells were plated with varying concentrations of afatinib, neratinib, and poziotinib for 72 h. ERBB2^WT^ cells were plated in media supplemented with IL-3. Cell viability was determined using a tetrazolamine-based viability assay. Viability is represented as a percentage of the untreated control. The average mean ± SEM is shown.

Given the observed factor-independent growth and heightened ErbB2 activation, we assessed the ex vivo sensitivity of primary patient samples harboring ERBB2 R188C, P489L, and L1157R mutations to a panel of clinically relevant ErbB inhibitors that were available at the time of patient sample accrual. Our panel consisted of reversible ErbB family inhibitors (i.e., erlotinib, gefitinib, and lapatinib) and two investigational irreversible inhibitors (i.e., canertinib and pelitinib). Among the reversible inhibitors, erlotinib and gefitinib are currently FDA-approved for the treatment of NSCLC harboring exon-19 deletions and activating EGFR mutations, and lapatinib is approved for metastatic HER2^+^ breast cancer [2]. However, only lapatinib targets cancers that overexpress ERBB2/HER2; erlotinib and gefitinib act in cancers with mutated or overactive EGFR [2]. Upon patient sample procurement, leukemia cells were assayed for cell survival after incubation with one of four graded concentrations of a given drug and a third-order polynomial curve was fit per sample and inhibitor. An IC_50_ value was calculated for each inhibitor and compared with the median of the inhibitor IC_50_s for the overall patient sample cohort of similar four-point dose responses. Inhibitors for which the IC_50_ value was less than 20% of the cohort median were considered effective given historical inhibitor screening data [10]. Considering only members of the cohort with similar diagnoses (i.e., acute myeloid or lymphoid leukemias) did not change the result. On the whole, we saw little to no sensitivity to these reversible ErbB inhibitors in our primary patient samples (Fig. 2a). While this result may be expected for the EGFR inhibitors erlotinib and gefitinib, it was surprising to observe a minimal response with lapatinib.

Our ErbB2-mutant-transformed Ba/F3 cell lines were also insensitive to erlotinib and gefitinib (Supplementary Fig. 4A, Supplementary Table 2). However, we saw some activity of lapatinib. Of the three mutants, ERBB2^P489L^-transformed Ba/F3 cells showed modest sensitivity to lapatinib with an IC_50_ of 27.1 nM followed by ERBB2^R188C^ and ERBB2^L1157R^-transformed cells with an IC_50_ of 38.0 and of 53.8 nM, respectively. In each case, ERBB2^WT^ Ba/F3 cells grown in IL-3-supplemented media were insensitive to all inhibitors.

We observed a trend toward increased sensitivity in patient samples to pelitinib and canertinib, especially against the ERBB2^L1157R^ mutation with an IC_50_ of 0.6 and 2.2 µM respectively. The patient with ERBB2^R188C^ showed higher sensitivity to canertinib with an IC_50_ of 2.9 µM in comparison with the patient with ERBB2^P489L^. However, on the whole, the response to these inhibitors in patient samples was limited in comparison with their response observed with ERBB2-mutant-expressing Ba/F3 cell lines. All ERBB2-mutant-expressing Ba/F3 cell lines were sensitive to irreversible ErbB inhibitors. Pelitinib potently inhibited the proliferation of the ERBB2-mutant-expressing Ba/F3 lines with IC_50_ values of 18.7, 10.9, and 19.3 nM for ERBB2^R188C^, ERBB2^P489L^, and ERBB2^L1157R^, respectively. Mutant lines exhibited even greater sensitivity canertinib, with IC_50_ values ranging from 3.3 to 8.4 nM (Supplementary Fig. 4B, Supplementary Table 2).

The sensitivity differences observed between primary samples and cell line models could potentially result from additional mutations. We observed that the pediatric patient with ERBB2^R188C^ had a MAP3K10^P168Q^ co-mutation, while the pediatric patient with ERBB2^P489L^ harbored additional mutations in MAP4K1^P422L^ and CSF3R^M696T^. A FLT1^I623V^ mutation was found in the patient with ERBB2^L1157R^ (Supplementary Fig. 1). While the functional significance of these mutations is undetermined, MAP3K10 and MAP4K1 lead to activation of the JNK pathway, which could contribute to leukemia cell growth downstream of ERBB2 [11]. Similarly, CSF3R and FLT1 could activate downstream MAPK and JAK/STAT signaling, further facilitating leukemogenesis [10]. In addition, the possible subclonal status of the ERBB2^R188C^ mutation with a VAF of 29% with a disease burden of 91% blasts within the marrow may also explain the limited sensitivity observed to ErbB inhibitors.

Given the increased potency observed for the investigational irreversible ERBB2 inhibitors pelitinib and canertinib, we expanded drug profiling to evaluate the response of our ErbB2-mutant-transformed Ba/F3 lines to the current FDA-approved irreversible ErbB inhibitors afatinib and neratinib. These second-generation inhibitors irreversibly inhibit both EGFR and ErbB2 and have shown potency against HER2^+^ breast cancer [2]. Neratinib is also particularly well suited to target the EGFR^T790M^ gatekeeper mutation that mediates resistance in NSCLC [2]. Treatment of mutant-transformed ERBB2 Ba/F3 cells with either afatinib or neratinib potently inhibited cell growth with IC_50_ values ranging 1.6 to 3.2 and 0.8 to 1.8 nM, respectively (Fig. 2c). In addition, a recent pan-cancer analysis revealed that poziotinib, an irreversible pan-ErbB family inhibitor, has broad antitumor effects in multiple ERBB2-mutant cancer types independent of the mutation location [12]. This compound was equally robust in inhibiting growth in our Ba/F3 lines with an IC_50_ of 1.1 nM for ERBB2^R188C^, 1.9 nM for ERBB2^P489L^, and 0.7 nM for ERBB2^L1157R^ (Fig. 2c, Supplementary Table 2). Since ex vivo small-molecule inhibitor screening requires the use of fresh patient samples, we cannot comment on how patients with ERBB2 R188C, P489L, or L1157R would respond to afatinib, neratinib, and poziotinib. Nonetheless, our small-molecule inhibitor data in Ba/F3 cells show that our ERBB2 point mutants are generally sensitive to irreversible ErbB inhibitors in the nanomolar range. These findings are in line with previous studies that also show increased efficacy of irreversible over reversible inhibitors against ERBB2 point mutations [4, 12].

Lastly, we evaluated the efficacy of trastuzumab in our mutant-transformed Ba/F3 cell lines. Compared with the cytoplasmic mutant ERBB2^L1157R^, both extracellular domain mutants, ERBB2^R188C^ and ERBB2^P489L^, demonstrated higher sensitivity to trastuzumab with an IC_50_ of 1.2 and 0.6 nM, respectively. However, neither mutant reached an IC_90_ following treatment with trastuzumab (Supplementary Fig. 4C, Supplementary Table 2).

The advent of next-generation sequencing technologies has enabled the identification of ERBB2/HER2 point mutations in various cancers. However, their functional characterization and oncogenic potential has yet to be rigorously investigated. Despite the paucity of ERBB2 expression in hematopoietic tissues, in this study, we discovered three oncogenic ERBB2 point mutations—R188C, P489L, and L1157R—in patients with acute leukemia. Stable expression of these mutations in Ba/F3 cells provided a proliferative advantage and enabled increased activation of ErbB2. ERBB2^R188C^ is located within subdomain II of the extracellular domain, a region that is characterized by 11 disulfide bonds. Based upon data from previously reported mutations in ERBB2 (C311R and C334S) and CSF2Rβ (R1461C), we speculate that R188C induces constitutive receptor activation via formation of disulfide bonds between cysteine residues that stabilize the mutant receptor [13, 14]. In the case of mutations P489L and L1157R, these mutations may destabilize the inactive receptor, similar to the EGFR^L858R^ in lung cancer [15], culminating in active receptor molecules that contribute to the underlying leukemia. Particularly, the loss of proline with mutant P489L could introduce flexibility into the protein that destabilizes the receptor and enables it to readily adopt the activated form. This could explain why it predominately resides in the phosphorylated form as evident from our immunoblot results.

Selective inhibition with irreversible ErbB inhibitors attenuated proliferation and induced cell death of our mutant-transformed ERBB2 Ba/F3 cells. It should be noted that the current clinical indication for the approved ERBB2 inhibitors afatinib, lapatinib, and neratinib is limited to cancers with ERBB2 amplification and overexpression. No small-molecule inhibitor is currently approved for cancers harboring oncogenic ERBB2 point mutations.

In aggregate, we report three functional ERBB2 mutants in leukemia that are highly sensitive to irreversible ErbB inhibitors in our cytokine-independent cellular assay. Whether these mutations drive leukemia or mediate secondary resistance is currently unknown. Nonetheless, our data suggest that these mutations have the potential to contribute to leukemogenesis and highlight the importance of investigating rare, yet clinically targetable mutations, as such studies further our ability to develop precise and individualized treatment regimens for a subset of patients with leukemia.

## Supplementary information

Supplemental Methods File

Supplemental Table 1

Supplemental Table 2

Supplemental Figure 1

Supplemental Figure 2

Supplemental Figure 3

Supplemental Figure 4
