# Supplementary material for: ERBB2/HER2 mutations are transforming and therapeutically targetable in leukemia
Source: Leukemia. 2020 May 4;34(10):2798–804. doi: 10.1038/s41375-020-0844-7 (PMC7515826; doi:10.1038/s41375-020-0844-7)
Supplement: Supplementary file 1 — Supplemental Methods File [file 41375_2020_844_MOESM1_ESM.docx]

**Supplemental Methods, Figure Legends, & Tables**

***ERBB2/HER2 mutations are transforming and therapeutically targetable in leukemia***

Sunil K. Joshi^1,2,3,^ **^*^**, Jamie M. Keck^1,4,^ **^*^**, Christopher A. Eide^1,3^, Daniel Bottomly^1^, Elie Traer^1,3,5^, Jeffrey W. Tyner^1,3,5^, Shannon K. McWeeney^1,6^, Cristina E. Tognon^1,3, §^, Brian J. Druker^1,3,5, §^

^1^Knight Cancer Institute, Oregon Health & Science University, Portland, OR

^2^Department of Physiology & Pharmacology, School of Medicine, Oregon Health & Science University, Portland, OR

^3^Division of Hematology & Medical Oncology, Department of Medicine, Oregon Health & Science University, Portland, OR

^4^Center for Spatial Systems Biomedicine, Oregon Health & Science University, Portland, OR

^5^Department of Cell, Development, & Cancer Biology, Oregon Health & Science University, Portland, OR

^6^Division of Bioinformatics and Computational Biology, Department of Medical Informatics and Clinical Epidemiology, Oregon Health & Science University, Portland, OR

*****These authors equally contributed to this work.

**TABLE OF CONTENTS:**

**SUPPLEMENTAL MATERIALS & METHODS………………………………………...…………..3-7**

**SUPPLEMENTAL FIGURE LEGENDS……………………………………………………….…….8-9**

**SUPPLEMENTAL TABLE LEGENDS…………………………………………………………….…10**

**REFERENCES………………………………………………………………………………...………..11**

**METHODS:**

***Sequencing of patient samples***

Clinical specimens were collected with informed consent from patients according to a biorepository research protocol approved by the Oregon Health & Science University institutional review board (IRB 4422; NCT01728402). Deep sequencing was performed on 1,862 kinase and kinase-associated genes using a custom capture library as described previously^1, 2^. A total of 185 patient samples were sequenced: 96 acute myeloid leukemia (AML), 51 acute lymphoblastic leukemia (ALL), and 38 myeloproliferative neoplasms (MPNs). It should be noted that the capture library is focused on kinase and kinase-associated genes, and so there may be other mutations present that were not assessed using this platform.

ERBB2 mutations were confirmed via Sanger sequencing genomic DNA using the following primers: ERBB2^R188C^ Forward 5’—GCCCCAAGGGAAGCAGAAGGTG—3’, Reverse 5’—CCCCAGAGATGAAGAGGCACAGGG—3’; ERBB2^P489L^ Forward 5’—CCTGTGGGAAGCTTTGGGCCTG—3’, Reverse 5’—GACGGGCAGTCTGCACAAGTCC—3’; ERBB2^L1157R^ Forward 5’—AGCGGTACAGTGAGGACCCCAC—3’, Reverse 5’—TTCCCCTCTCAGGCCAGCTTCC—3’.

***Functional drug screening-guided variant prioritization***

Primary patient mononuclear cells were screened ex vivo using an established panel of small-molecule inhibitors as previously described^3^. Genomic variants were prioritized based on the HitWalker algorithm, which integrates genomic information with functional screening (i.e., ex vivo small-molecule inhibitor screening) to identify oncogenic targets as described previously^4, 5^. First scores were assigned to genes based on the small-molecule inhibitor profiles in relation to putative gene-targets^6^. Based on the distribution of these gene scores, the top genes were chosen as ‘seeds’. A network propagation approach was used to rank genes in the STRING^7^ network with respect to seed proximity. Using their relative ranks, the top ten putative gene mutations were chosen for further inspection.

***Affymetrix exon microarray***

cDNA was prepared from available patient RNA. Samples were amplified and labeled using the Affymetrix WT Terminal Labeling kit (ThermoFisher Scientific Inc.) per manufacturer instructions.

The exon microarray data was processed using the ‘oligo’ package^8^. Expression values for the ‘core’ probesets were computed using the robust-multiarray average (RMA) method^9^. The probeset detected above background (PSDABG) approach was used to calculate a p-value indicating whether a given probeset (roughly an exon) was more expressed than background. In practice this was done by computing p-values for each probe and sample using the DABG approach^10^ with the resulting p-values summarized to the probeset level using Fisher’s method. These data are available from GEO (GSE42731).

***Cell culture***

Ba/F3 cells were maintained in RPMI (Life Technologies Inc.) supplemented with 10% FBS, 2% L-glutamine, 1% penicillin/streptomycin, 0.1% amphotericin B and 15% WEHI-3B-conditioned medium (a source of IL-3). NIH 3T3 cells (ATCC) were grown in DMEM (Life Technologies Inc.) supplemented with 10% calf serum (Atlanta Biologicals).

***Retroviral transduction & downstream assays***

Wild-type and mutant ERBB2 cDNA constructs were ectopically expressed in Ba/F3 and NIH 3T3 cells using a Gateway-modified pMX-IRES-GFP puro retroviral vector (Cell BioLabs Inc.)^2, 11^. All ERBB2 mutations were introduced by site-directed mutagenesis using the QuikChange II XL kit (Agilent Technologies Inc.). The following primers were used for mutagenesis: ERBB2^R188C^ Forward 5’—GCAGGCCCGAGAGCAGTTGGTGTCTATCA—3’, Reverse 5’—

TGATAGACACCAACTGCTCTCGGGCCTGC—3’; ERBB2^P489L^ Forward 5’—GCAGAGCTTGGTGCAGGTTCCGAAAGAGC—3’, Reverse 5’—GCTCTTTCGGAACCTGCACCAAGCTCTGC—3’; ERBB2^L1157R^ Forward 5’—

GGGCAGCAGGCCGAGGGCCCTCT—3’, Reverse 5’—AGAGGGCCCTCGGCCTGCTGCCC

—3’.

Cells were then sorted using a FACSAria flow cytometer (BD Biosciences Inc.) for equivalent, low levels of GFP expression to avoid the known transformative effects of highly overexpressed ErbB2 levels. Ba/F3 cell culture model provides a well-established model to study kinase mutations^12^. For IL-3 withdrawal studies, Ba/F3 cell lines were seeded at a density of 5 x 105 cells/mL, monitored by Guava Viacount assay (Millipore Inc.) and expanded as described previously^2, 11^. NIH 3T3 lines expressing ERBB2 constructs were grown as a monolayer until confluence, and images were obtained using a Leica DM IL LED microscope (Leica Microsystems Inc.). All were experiments were performed twice with consistent results.

***Immunoblotting***

Immunoblot analysis was performed on serum starved (0.1% BSA RPMI) Ba/F3 cells as described previously^1^. Following overnight serum starvation, 10-15 million WT and ERBB2 mutant Ba/F3 cells were spun down and lysed with 200 μL of Cell Lysis Buffer (Cell Signaling Technologies Inc.) containing a Complete Mini Protease Inhibitor Cocktail Tablet, Phosphatase Inhibitor Cocktail 2, and Phenylmethanesulfonyl Fluoride (PMSF) solution (Sigma-Aldrich Inc.) and clarified by centrifugation at 14,000 *g*, 4 °C for 15 minutes. 50 μg of each protein lysate was loaded on NuPAGE 4-12% Bis-Tris gradient gels (Thermo Fisher Scientific Inc.), transferred on Immobilon-FL PVDF membranes (Millipore Inc.), blocked for 1 hour, and incubated with primary antibody overnight. The primary antibodies used were rabbit anti-ErbB2 (Cell Signaling Technology; #2242), anti-phospho-ErbB2 (#2243) and mouse anti-GAPDH (ThermoFisher; #AM4300). Following overnight incubation with primary antibody at 4 °C, the membranes were washed and probed with fluorescent IRDye 800CW goat anti-rabbit IgG and IRDye 680RD Goat anti-mouse IgG antibodies (1:15,000; LI-COR Biosciences). The membranes were then imaged with the Odyssey Infrared Imaging System (LI-COR Biosciences).

***Gene expression profiling***

Isolated RNA (Qiagen) from serum starved ERBB2-expressing Ba/F3 cell lines (n=3 per cell line) was evaluated for nCounter analysis using the Pan-Cancer Human Pathways codeset (Nanostring Technologies Inc.).

***Inhibitor studies***

Ba/F3 cell lines or primary leukemia cells were seeded in 384-well plates, incubated with the indicated concentrations of inhibitor for 72 hours, and assessed for cell survival using a methanethiosulfonate (MTS)-based assay (Promega) as previously described^3, 6^. The final concentration of DMSO was ≤0.1% in all wells. Five technical replicates were plated for all Ba/F3 cell lines. Small-molecule inhibitors were purchased from LC Laboratories Inc. and Selleck Chemicals. Herceptin was purchased from MedChemExpress. Analysis for the cell line was completed via GraphPad Prism version 8.0. For the primary leukemia cells, raw absorbance values were first adjusted to a reference blank and then normalized to untreated control wells to form a measure of normalized viability expressed as a percentage. The concentration values were log transformed and curves were fit to these data using a 3^rd^ degree polynomial. The IC_50_ was defined as the estimated concentration where the normalized viability was equal to 50%. An IC_50_ value was calculated for each inhibitor and compared with the median of the inhibitor IC_50_s for the overall patient sample cohort of similar 4-point dose-responses. Inhibitors for which the IC_50_ value was less than 20% of the cohort median were considered effective given historical inhibitor screening data^1^. It should be noted that *ex vivo* inhibitor testing can only be done on fresh patient samples at the time of sample procurement. Frozen samples have compromised viability, which confounds interpretation of the results.

**SUPPLEMENTAL FIGURE LEGENDS:**

**Supplemental Figure 1: ERBB2 point mutations represent important targets in leukemia using the HitWalker algorithm that integrates deep sequencing data with functional screening.** Hitwalker diagrams are shown for patient: 08-00053 expressing ERBB2^R188C^ (**A**), 09-00076 expressing ERBB2^P489L^ (**B**), 11-00319 expressing ERBB2^L1157R^ (**C**). The functional targets (acquired from inhibitor screening data) are shown in red, and mutated genes are shown in blue. Each mutated gene is assigned a rank based on predicted association with one or more putative drug targets. Networks are drawn using a Steiner Tree approximation^13^ with the seed genes in this context being the union of the functional targets and mutations.

**Supplemental Figure 2:** **3D mapping of ERBB2 point mutations.** **A.** Gene schematic of ERBB2 with location of point mutations indicated. **B.** Relative positions of native ERBB2 amino acids (i.e., R188, P489, and L1157) for the mutations studied are highlighted using PyMOL. Since the ERBB2 receptor is not fully crystallized, the location of L1157 is approximated.

**Supplemental Figure 3: Summary of Affymetrix exon microarray expression for ERRB2.**  **A-B.** Expression was determined using the probeset detected above background (PSDABG) metric, which measures whether a given ERBB2 exon probeset signal was detected at significantly higher intensity than background regions with similar GC content^10^. Our analysis suggests that a majority of the ERBB2 exons are expressed above background for these samples. The RefSeq gene models for ERRB2 are provided relative to hg19 (bottom track). Each high-confidence probeset was tested for whether its expression was greater than background. A red line tracing the -log10(P-values) from this test is plotted in the middle track with the dot-dash line indicating the nominal 0.05 p-value threshold. If the given probesets would still be significant after FDR correction they are indicated by an '*'. The top track shows the probeset-level robust multi-array average (RMA) expression values with the dashed line indicating the median across all probesets for that sample. Only the high-confidence probesets are displayed here for clarity.

**Supplemental Figure 4: ERBB2 mutant-transformed Ba/F3 cell lines display minimal sensitivity to reversible ErbB inhibitors.** **A.** FDA-approved reversible ErbB inhibitors erlotinib, gefitinib, and lapatinib were tested. Lapatinib showed modest sensitivity against ERBB2 mutations. **B.** All ERBB2-transformed Ba/F3 cells were sensitive to irreversible inhibitors pelitinib and canertinib at low nanomolar concentrations. Higher sensitivity was observed with canertinib. **C.** Both extracellular domain ERBB2 mutants, R188C and P489L, demonstrated an increased response to trastuzumab. The trastuzumab antibody binds ERBB2 at the extracellular domain IV where it interacts with its partnering ErbB receptors, and therefore it would not be expected to effectively inhibit the cytoplasmic mutant, ERBB2^L1157R^, which plateaued after reaching its IC_50_ of 8.7 nM. Cell viability was determined using a tetrazolamine-based viability assay. Viability is represented as a percentage of the untreated control. The mean of five replicates is plotted, along with the SEM.

**SUPPLEMENTAL TABLE LEGENDS:**

**Supplemental Table 1:** Available clinical and mutation information on patients harboring ERBB2 point mutations. VAF from patient bone marrow aspirate is provided. These point mutations were checked in COSMIC and ExAC to determine if they were previously reported. Hitwalker ranking is provided. Of note, the higher disease burden in the bone marrow for all three patients (ranging from 72-100% blasts) makes it difficult to determine whether the 52% VAF for P489L and the 46% VAF for L1157R are heterozygous somatic or germline mutations. The R188C mutation, with a 29% VAF, is most likely somatic and potentially a prominent subclone within the leukemia, with approximately 60% of the 91% blasts harboring a heterozygous variant. PB, Peripheral Blood; BM, Bone Marrow; VAF, Variant Allele Frequency; CRLF2, Cytokine Receptor-like Factor 2.

**Supplemental Table 2:** Summary of IC_50_ values for inhibition of ERBB2 mutant-transformed Ba/F3 cell growth by small-molecule inhibitors calculated from data presented in Figure 2C and Supplemental Figure 4.

**REFERENCES:**

1. Maxson, J.E., J. Gotlib, D.A. Pollyea, A.G. Fleischman, A. Agarwal, C.A. Eide, et al., *Oncogenic CSF3R mutations in chronic neutrophilic leukemia and atypical CML.* N Engl J Med, 2013. **368**(19): p. 1781-90. PMC3730275

2. Maxson, J.E., M.A. Davare, S.B. Luty, C.A. Eide, B.H. Chang, M.M. Loriaux, et al., *Therapeutically Targetable ALK Mutations in Leukemia.* Cancer Res, 2015. **75**(11): p. 2146-50. PMC4453002

3. Tyner, J.W., C.E. Tognon, D. Bottomly, B. Wilmot, S.E. Kurtz, S.L. Savage, et al., *Functional genomic landscape of acute myeloid leukaemia.* Nature, 2018. **562**(7728): p. 526-531. PMC6280667

4. Bottomly, D., B. Wilmot, J.W. Tyner, C.A. Eide, M.M. Loriaux, B.J. Druker, et al., *HitWalker: variant prioritization for personalized functional cancer genomics.* Bioinformatics, 2013. **29**(4): p. 509-10. PMC3570211

5. Maxson, J.E., M.L. Abel, J. Wang, X. Deng, S. Reckel, S.B. Luty, et al., *Identification and Characterization of Tyrosine Kinase Nonreceptor 2 Mutations in Leukemia through Integration of Kinase Inhibitor Screening and Genomic Analysis.* Cancer Res, 2016. **76**(1): p. 127-38. PMC4703549

6. Tyner, J.W., W.F. Yang, A. Bankhead, 3rd, G. Fan, L.B. Fletcher, J. Bryant, et al., *Kinase pathway dependence in primary human leukemias determined by rapid inhibitor screening.* Cancer Res, 2013. **73**(1): p. 285-96. PMC3537897

7. Szklarczyk, D., J.H. Morris, H. Cook, M. Kuhn, S. Wyder, M. Simonovic, et al., *The STRING database in 2017: quality-controlled protein-protein association networks, made broadly accessible.* Nucleic Acids Res, 2017. **45**(D1): p. D362-D368. PMC5210637

8. Carvalho, B.S. and R.A. Irizarry, *A framework for oligonucleotide microarray preprocessing.* Bioinformatics, 2010. **26**(19): p. 2363-7. PMC2944196

9. Irizarry, R.A., B. Hobbs, F. Collin, Y.D. Beazer-Barclay, K.J. Antonellis, U. Scherf, et al., *Exploration, normalization, and summaries of high density oligonucleotide array probe level data.* Biostatistics, 2003. **4**(2): p. 249-64.

10. Clark, T.A., A.C. Schweitzer, T.X. Chen, M.K. Staples, G. Lu, H. Wang, et al., *Discovery of tissue-specific exons using comprehensive human exon microarrays.* Genome Biol, 2007. **8**(4): p. R64. PMC1896007

11. Watanabe-Smith, K., C. Tognon, J.W. Tyner, J.P. Meijerink, B.J. Druker, and A. Agarwal, *Discovery and functional characterization of a germline, CSF2RB-activating mutation in leukemia.* Leukemia, 2016. **30**(9): p. 1950-3. PMC5014660

12. Warmuth, M., S. Kim, X.J. Gu, G. Xia, and F. Adrian, *Ba/F3 cells and their use in kinase drug discovery.* Curr Opin Oncol, 2007. **19**(1): p. 55-60.

13. Sadeghi, A. and H. Frohlich, *Steiner tree methods for optimal sub-network identification: an empirical study.* BMC Bioinformatics, 2013. **14**: p. 144. PMC3674966
