# Supplementary material for: ERBB2/HER2 mutations are transforming and therapeutically targetable in leukemia
Source: Leukemia. 2020 May 4;34(10):2798–804. doi: 10.1038/s41375-020-0844-7 (PMC7515826; doi:10.1038/s41375-020-0844-7)
Supplement: Supplementary file 2 — Supplemental Table 1 [file 41375_2020_844_MOESM2_ESM.docx]

| **Table 1: Available Patient & Mutation Information** | | | | | | | | | | |
| --- | --- | --- | --- | --- | --- | --- | --- | --- | --- | --- |
| **Specimen ID** | **Diagnosis** | **Specific Diagnosis** | **Sex** | **Pediatric/Adult**  **(Age & Karyotype)** | **Blast %** | **ERBB2 Mutation** | **BM VAF** | **COSMIC Status** | **ExAC Frequency** | **HitWalker Ranking** |
| 08-00053 | AML | AMML with MLL rearrangement | F | Pediatric  (Age: 5  Karyotype: 46,XX,t(10;11)(p?11.2;q?23)[20]) | PB: 59  BM: 91 | **R188C** | 0.29 | No | 0.00003298 | 2 |
| 09-00076 | ALL | B-ALL, CRLF2^+^ | M | Pediatric  (unknown) | PB: 75  BM: 100 | **P489L** | 0.52 | Yes | 0.0007738 | 6 |
| 11-00319 | AML | NPM1^+^, FLT3-ITD^+^ | F | Adult  (Age: 66  Karyotype: 46,XX,t(1;15)(p32;q15)[19]/46,XX[1]) | PB: 45  BM: 72 | **L1157R** | 0.46 | No | 0.00007937 | 5 |

PB, Peripheral Blood; BM, Bone Marrow; VAF, Variant Allele Frequency

CRLF2, Cytokine Receptor-like Factor 2
