# Supplementary material for: ERBB2/HER2 mutations are transforming and therapeutically targetable in leukemia
Source: Leukemia. 2020 May 4;34(10):2798–804. doi: 10.1038/s41375-020-0844-7 (PMC7515826; doi:10.1038/s41375-020-0844-7)
Supplement: Supplementary file 3 — Supplemental Table 2 [file 41375_2020_844_MOESM3_ESM.docx]

| **Table 2: Summary of inhibitor screening on mutant-transformed Ba/F3 cell lines** | | | | |
| --- | --- | --- | --- | --- |
| **Inhibitors** | **IC_50_ (nM)** | | | |
|  | **ERBB2^WT^** | **ERBB2^R188C^** | **ERBB2^P489L^** | **ERBB2^L1157R^** |
| Erlotinib | 727.4 | 500.0 | 390.6 | 396.3 |
| Gefitinib | >1000 | 526.4 | 717.8 | ~ 539.0 |
| Lapatinib | 694.1 | 38.0 | 27.1 | 53.8 |
| Afatinib | 628.6 | 2.6 | 1.6 | 3.2 |
| Canertinib | 661.3 | 8.4 | 3.3 | 6.4 |
| Neratinib | 573.9 | 1.7 | 0.8 | 1.8 |
| Pelitinib | 474.1 | 18.7 | 10.9 | 19.3 |
| Poziotinib | >1000 | 1.1 | 1.9 | 0.7 |
| Trastuzumab | >1000 | 1.2 | 0.6 | 8.7 |
