# Supplementary figures and images for: ERBB2/HER2 mutations are transforming and therapeutically targetable in leukemia
Source: Leukemia. 2020 May 4;34(10):2798–804. doi: 10.1038/s41375-020-0844-7 (PMC7515826; doi:10.1038/s41375-020-0844-7)

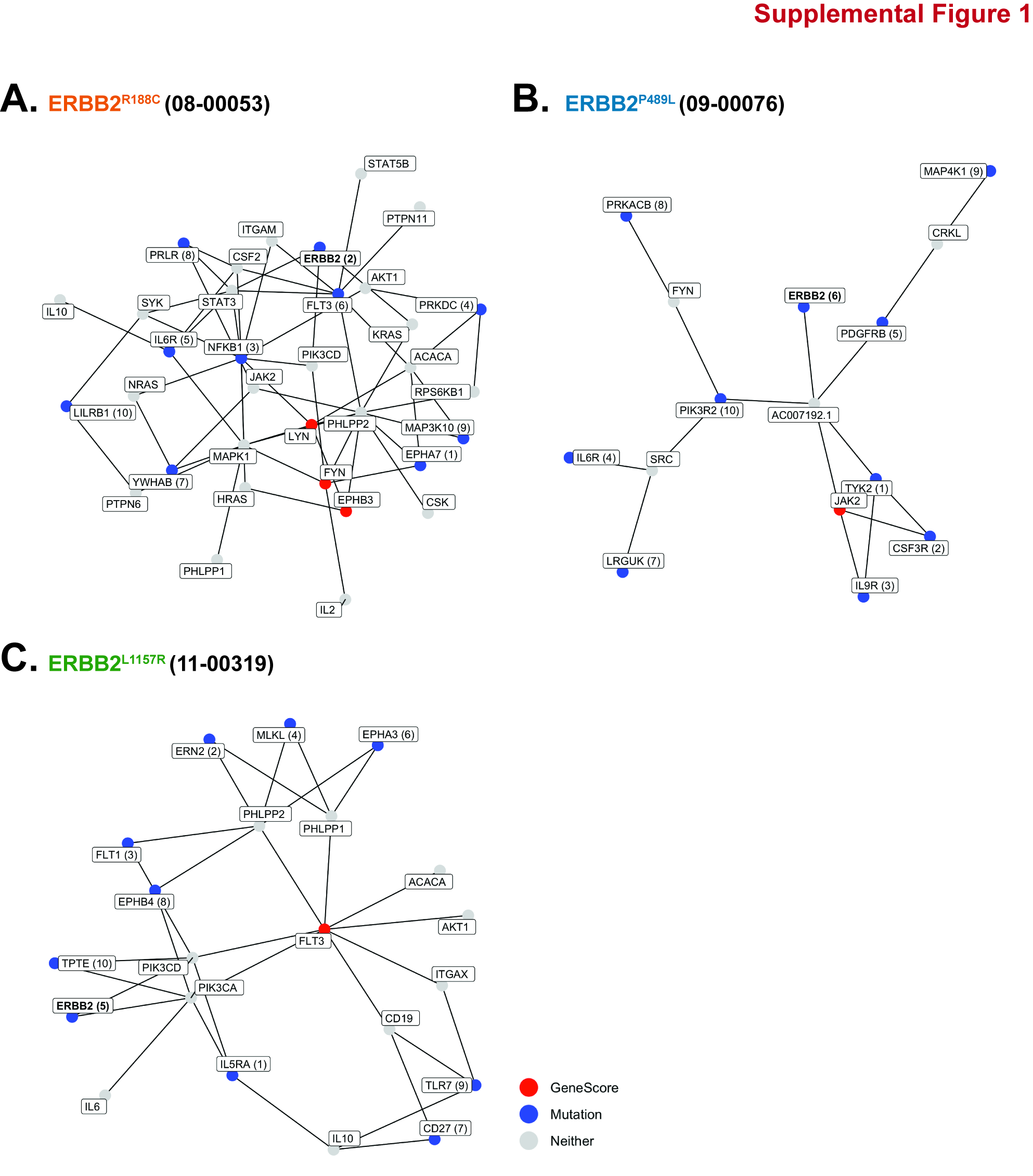

Supplement: Supplementary file 4 — Supplemental Figure 1 [file 41375_2020_844_MOESM4_ESM.tif]

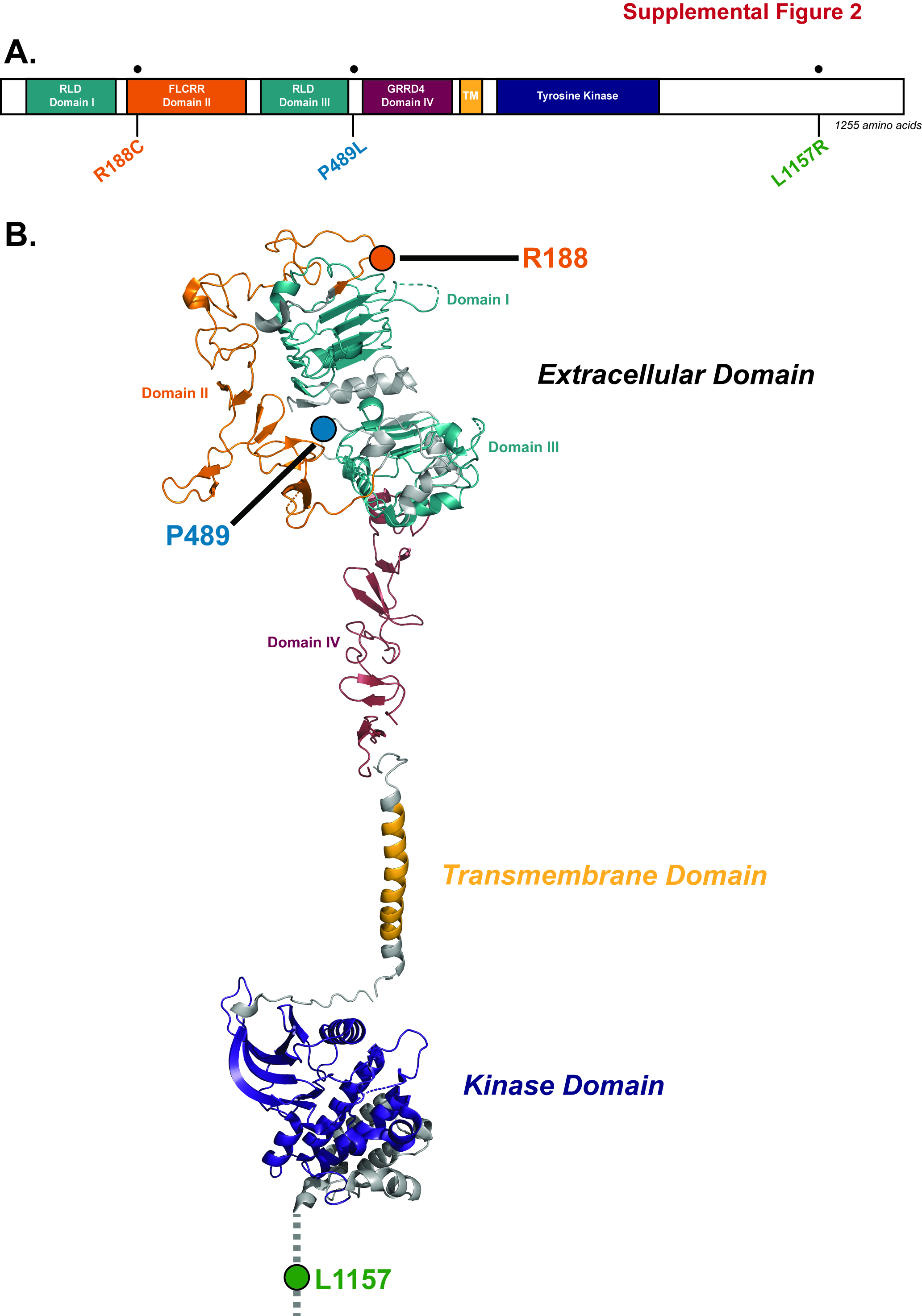

Supplement: Supplementary file 5 — Supplemental Figure 2 [file 41375_2020_844_MOESM5_ESM.tif]

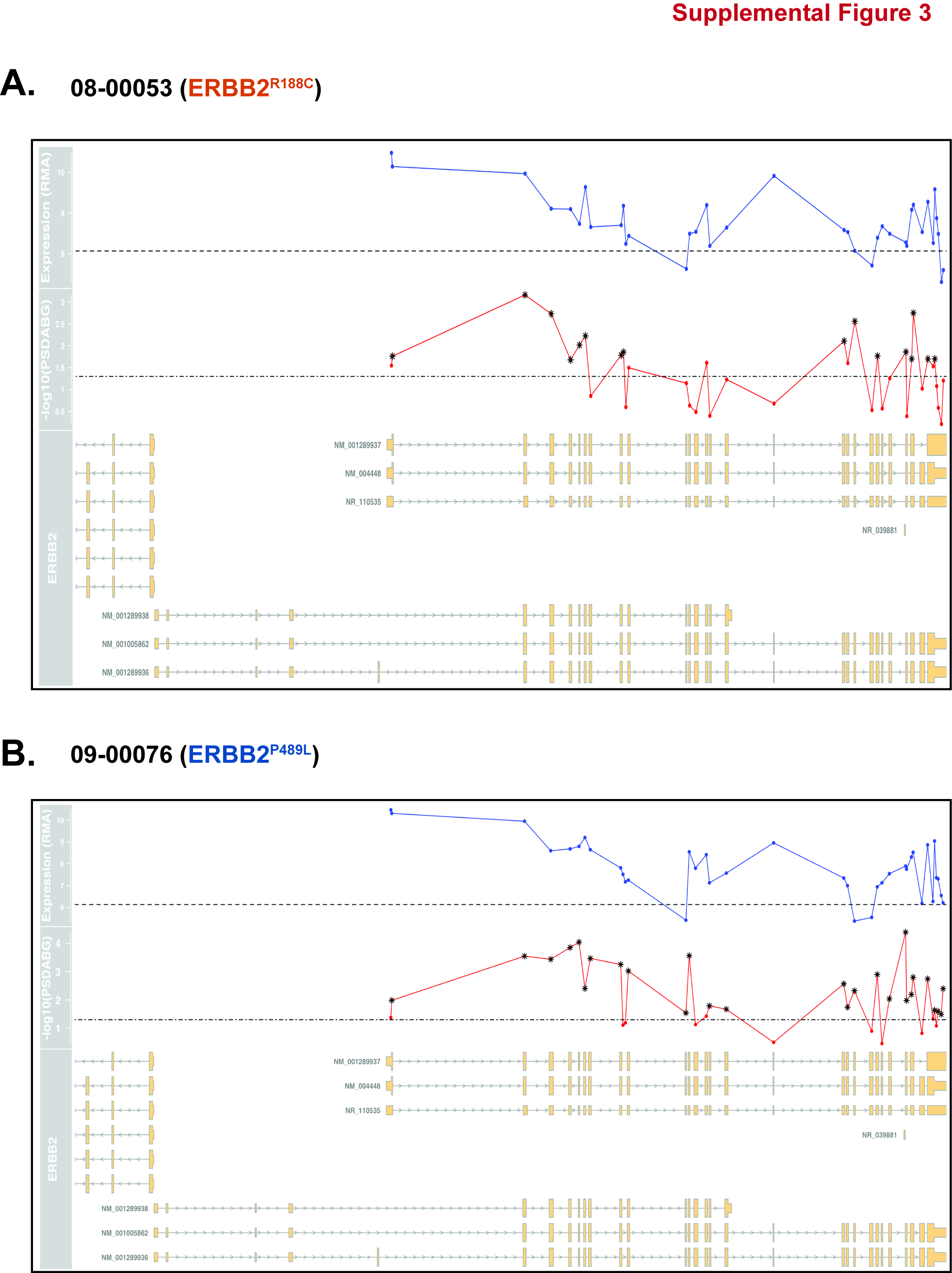

Supplement: Supplementary file 6 — Supplemental Figure 3 [file 41375_2020_844_MOESM6_ESM.tif]

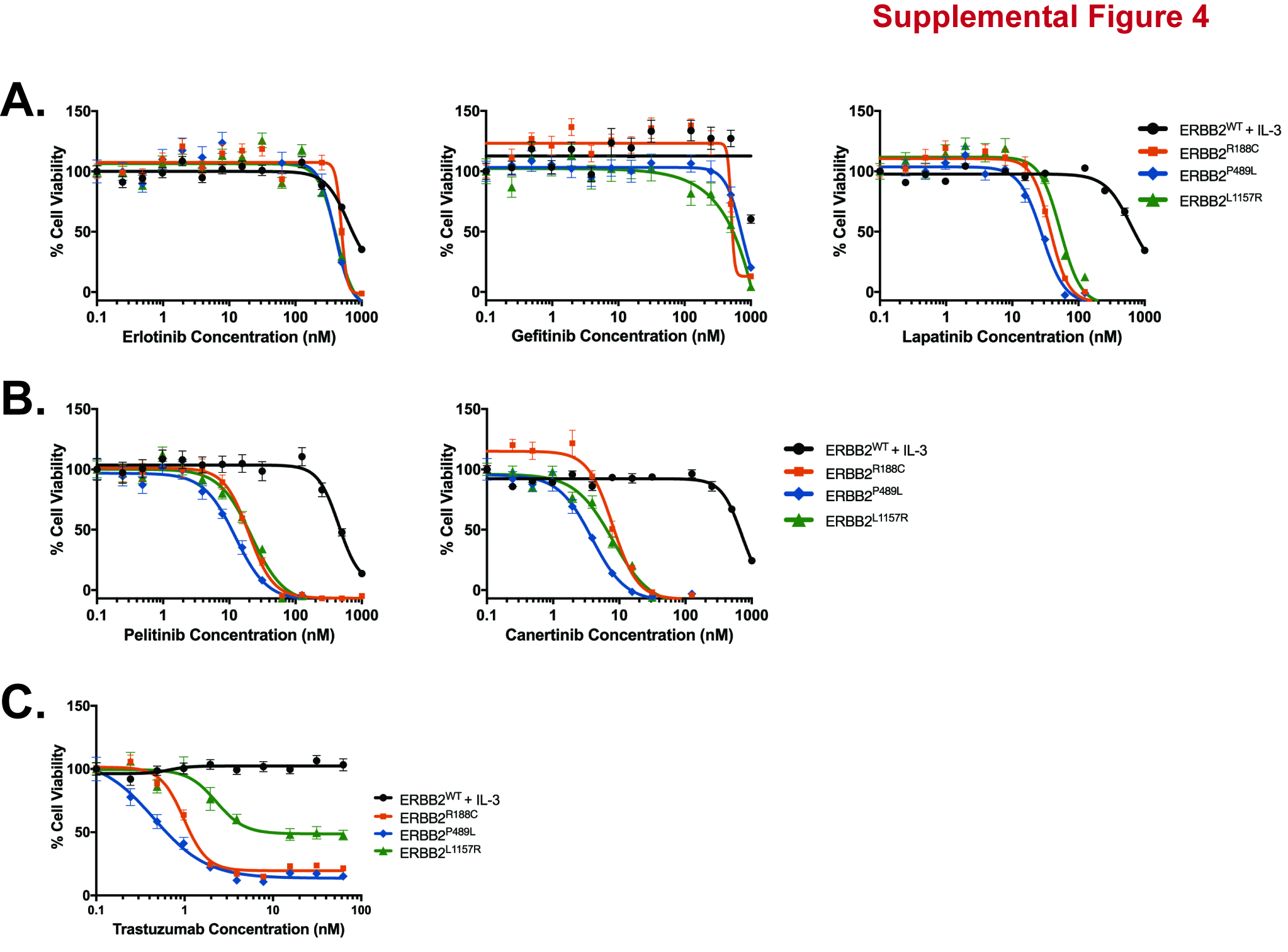

Supplement: Supplementary file 7 — Supplemental Figure 4 [file 41375_2020_844_MOESM7_ESM.tif]
